# Simple method for predicting muscle volume loss using geriatric nutritional risk index in hepatocellular carcinoma patients

**DOI:** 10.1002/jcsm.13268

**Published:** 2023-05-19

**Authors:** Atsushi Hiraoka, Hideko Ohama, Fujimasa Tada, Yoshiko Fukunishi, Emi Yanagihara, Kanako Kato, Masaya Kato, Hironobu Saneto, Hirofumi Izumoto, Hidetaro Ueki, Takeaki Yoshino, Shogo Kitahata, Tomoe Kawamura, Taira Kuroda, Yoshifumi Suga, Hideki Miyata, Masashi Hirooka, Masanori Abe, Bunzo Matsuura, Tomoyuki Ninomiya, Yoichi Hiasa

**Affiliations:** ^1^ Gastroenterology Center Ehime Prefectural Central Hospital Matsuyama Japan; ^2^ Department of Gastroenterology and Metabology Ehime University Graduate School of Medicine Toon Japan

## Introduction

The liver is a central organ that controls metabolic nutrition, whereas tumour burden and hepatic function are well‐known major prognostic factors in hepatocellular carcinoma (HCC) patients.^1^, ^2^ Nutritional status generally becomes worse with progression of hepatic function decline and conditions such as protein‐energy malnutrition (PEM) often complicated in liver cirrhosis (LC) patients.^3^ As a result of such a worsened status, muscle volume loss (MVL) often develops in chronic liver disease (CLD) patients.^4^ MVL has been recognized as an important prognostic factor in HCC patients treated either curatively or palliatively.^5^ However, special technologies, such as computer software for use with computed tomography (CT) or devices for bioelectrical impedance analysis (BIA), are generally needed for assessment of muscle volume; thus, many institutions have difficulties accessing such methods because of their expense. Previously, a nutritional assessment index termed geriatric nutritional risk index (GNRI),^6^ which is calculated with use of only serum albumin level, height and body weight, was developed.

The present study aimed to elucidate the clinical usefulness of GNRI as an easy nutritional assessment method using well‐known clinical factors to predict a high risk of MVL in CLD patients with HCC.

## Materials and methods

Four hundred forty two HCC patients, who underwent CT examinations performed at our hospital from January 2017 to June 2022 and within 1 month before starting treatment for HCC, were enrolled. None had a past history of HCC. Their records were kept in an institutional database and analysed in a retrospective manner.

### Basal liver disease

HCC was judged to be due to hepatitis C virus (HCV) in patients positive for anti‐HCV, whereas HCC due to hepatitis B virus (HBV) was determined in those positive for the HBV surface antigen.

### Assessments of liver function and nutritional status

For assessment of hepatic reserve function, Child–Pugh classification^7^ and albumin–bilirubin (ALBI) score^2^, ^8^ were used, along with modified ALBI (mALBI) grade, with ALBI grade 2 divided into two sub‐grades (2a and 2b) using an ALBI score of −2.27 as the cut‐off value.^9^ GNRI was utilized as an assessment tool for nutritional status using the following formula: GNRI score = 14.89 × serum albumin (g/dL) + 41.7 × present body weight (kg)/standard weight (kg). Furthermore, the formulas for calculating standard body weight were as follows: males, height (cm) − 100 − (height (cm) − 150)/4, and females, height (cm) − 100 − (height (cm) − 150)/2.5. The GNRI scores were separated into four status grades: normal (≥98), mild nutritional decline (<98 to 92), moderate nutritional decline (<92 to 82) and severe nutritional decline (<82).^6^

### Muscle volume evaluation and definition of muscle volume loss

CT results at the baseline examination, performed within 1 month of diagnosis of HCC, were used. The muscle area at the middle of the L3 level was evaluated using a Synapse Vincent 3D image analysis system, Version 5.5.0007 (FUJIFILM Corporation, Tokyo, Japan), by one of the authors (AH), with skeletal muscle index (SMI), determined based on muscle area (cm^2^), calculated using the middle of the L3 level/height (m)^2^. MVL was defined in males as ≤42 cm^2^/m^2^ and in females as ≤38 cm^2^/m^2^.^10^ The measuring process and results were confirmed by a different author (HO).

### Hepatocellular carcinoma diagnosis and treatment

HCC was diagnosed based on findings showing an increasing course of alpha‐fetoprotein (AFP), as well as results obtained with dynamic CT^11^ or magnetic resonance imaging (MRI),^12^, ^13^ and/or a pathological technique. Early‐stage HCC was defined based on the Milan criteria.^14^

This study was based on the Guidelines for Clinical Research issued by the Ministry of Health and Welfare of Japan, and all procedures were performed in accordance with the Declaration of Helsinki. Informed consent was obtained in the form of an opt‐out option. Those who chose to not participate were excluded from the study and their results are not shown.

### Statistical analysis

Median values (interquartile range [IQR]) are used to express continuous variables. For statistical analyses, Student's *t*‐test, Fisher's exact test, Mann–Whitney's *U* test, one‐way analysis of variance (ANOVA), Kruskal–Wallis's test, Pearson's test, receiver operating characteristic (ROC) curve analysis and area under the curve (AUC) were utilized.

*P* values < 0.05 were considered to indicate statistical significance. Easy R (EZR), Version 1.53 (Saitama Medical Center, Jichi Medical University, Saitama, Japan),^15^ a graphical user interface for R (The R Foundation for Statistical Computing, Vienna, Austria), was used for the statistical analyses.

## Results

### Cohort characteristics

The cohort characteristics are shown in *Table* *1*. Patients with MVL had older age (median: 78 vs. 72 years, *P* < 0.001), lower body mass index (BMI) (20.7 vs. 24.6 kg/m^2^, *P* < 0.001) and worse status for the Eastern Cooperative Oncology Group Performance Status (ECOG PS) (1:2:3:4 = 63:21:10:5:6 vs. 289:25:12:8:3, *P* < 0.001), Child–Pugh classification (A:B:C = 69:28:8 vs. 288:39:10, *P* < 0.001), mALBI grade (31:22:36:16 vs. 169:61:88:19, *P* < 0.001), frequency of HCC beyond the Milan criteria (50.5% vs. 38.0%, *P* = 0.031) and GNRI status (normal:mild:moderate:severe = 28:20:36:21 vs. 255:33:40:9, *P* < 0.001) (*Table* *2*).

**Table 1:** Clinical features of all 442 patients

Age, years (median^a^)	74 (67–80)
Gender, male (%)	321 (72.6%)
Body mass index, kg/m^2^ (median^a^)	23.7 (21.5–26.2)
ECOG PS, 0:1:2:3:4	352:46:22:13:9
Aetiology, HCV:HBV:HCV&HBV:alcohol:others	188:36:2:84:132
Child–Pugh class, A:B:C	357:67:18
Ascites (none:controllable:massive)	370:51:21
mALBI grade, 1:2a:2b:3	200:83:124:35
ALBI score (median^a^)	−2.52 (−2.05 to −2.87)
Milan criteria, beyond (%)	181 (41.0%)
Positive for MVI (%)	41 (9.3%)
Positive for EHM (%)	30 (6.8%)
MVL (%)	105 (23.8%)
GNRI nutritional status (normal:mild:moderate:severe)	283:53:76:30
GNRI score (median^a^)	102.3 (92.7–108.9)

Abbreviations: ECOG PS, Eastern Cooperative Oncology Group Performance Status; EHM, extra‐hepatic metastasis; GNRI, geriatric nutritional risk index; HBV, hepatitis B virus; HCV, hepatitis C virus; mALBI, modified albumin–bilirubin; MVI, major vessel invasion; MVL, muscle volume loss.

^a^ Median values in parentheses show interquartile range.

**Table 2:** Comparison of patients with and without muscle volume loss

	Negative for MVL (*n* = 337)	Positive for MVL (*n* = 105)	*P* value
Age, years (median^a^)	72 (67–79)	78 (71–85)	<0.001
Gender, male (%)	248 (73.6%)	73 (69.5%)	0.452
Body mass index, kg/m^2^ (median^a^)	24.6 (22.8–27.0)	20.7 (19.0–22.3)	<0.001
ECOG PS, 0:1:2:3:4	289:25:12:8:3	63:21:10:5:6	<0.001
Aetiology, HCV:HBV:HCV&HBV:alcohol:others	138:28:1:67:103	50:8:1:17:29	0.567
Child–Pugh class, A:B:C	288:39:10	69:28:8	<0.001
Ascites, none:controllable:massive	292:32:13	78:19:8	0.014
mALBI grade, 1:2a:2b:3	169:61:88:19	31:22:36:16	<0.001
ALBI score (median^a^)	−2.60 (−2.17 to −2.91)	−2.34 (−1.63 to −2.65)	<0.001
Milan criteria, beyond (%)	128 (38.0%)	53 (50.5%)	0.031
Positive for MVI (%)	26 (7.7%)	15 (14.3%)	0.053
Positive for EHM (%)	20 (5.9%)	10 (9.5%)	0.264
GNRI nutritional status (normal:mild:moderate:severe)	255:33:40:9	28:20:36:21	<0.001
GNRI score (median^a^)	104.8 (98.3–110.6)	89.7 (83.6–99.3)	<0.001

Abbreviations: ECOG PS, Eastern Cooperative Oncology Group Performance Status; EHM, extra‐hepatic metastasis; GNRI, geriatric nutritional risk index; HBV, hepatitis B virus; HCV, hepatitis C virus; mALBI, modified albumin–bilirubin; MVI, major vessel invasion; MVL, muscle volume loss.

^a^ Median values in parentheses show interquartile range.

### Relationships among muscle volume loss, modified albumin–bilirubin grade and geriatric nutritional risk index status

Along with worsened GNRI status, Child–Pugh class A (GNRI normal 95.8%, mild 75.5%, moderate 55.3%, severe 13.3%; *P* < 0.001) and mALBI grade 1/2a (normal 66.8%/17.3%, mild 13.2%/43.4%, moderate 5.3%/14.5%, severe 0%/0%; *P* < 0.001) also became worse, whereas the positive rate of MVL was increased (normal 9.9%, mild 37.7%, moderate 47.4%, severe 70.0%; *P* < 0.001) (*Table* *3*). For patients with normal GNRI status, those with MVL showed older age (77 vs. 72 years, *P* = 0.006) and lower BMI (21.9 vs. 25.1 kg/m^2^, *P* < 0.001), whereas there were no significant differences in regard to gender (*P* = 0.262), mALBI grade (*P* = 0.235), Child–Pugh class (*P* = 0.680), aetiology of liver disease (*P* = 0.536) or frequency of HCC beyond the Milan criteria (*P* = 1.00) (*Table* *S1*).

**Table 3:** Characteristics of patients based on geriatric nutritional risk index status

	Normal (*n* = 283)	Mild (*n* = 53)	Moderate (*n* = 76)	Severe (*n* = 30)	*P* value
Age, years (median^a^)	73 (67–79)	74 (70–83)	74 (67–83)	76 (69–84)	0.097
Gender, male (%)	208 (73.5%)	37 (69.8%)	56 (73.7%)	20 (66.7%)	0.826
Body mass index, kg/m^2^ (median^a^)	24.9 (23.0–27.3)	22.2 (20.624.0)	21.6 (19.7–23.8)	20.7 (19.2–21.4)	<0.001
ECOG PS, 0:1:2:3:4	244:24:10:3:2	40:11:1:0:1	52:9:7:8:0	16:2:4:2:6	<0.001
Aetiology, HCV:HBV:HCV&HBV:alcohol:others	113:24:1:55:90	22:6:0:7:18	41:5:1:16:13	12:1:0:6:11	0.390
Child–Pugh class, A:B:C	271:10:2	40:13:0	42:27:7	4:17:9	<0.001
Ascites, none:controllable:massive	263:15:5	42:10:1	54:15:7	11:11:8	<0.001
mALBI grade, 1:2a:2b:3	189:49:43:2	7:23:22:1	4:11:49:12	0:0:10:20	<0.001
ALBI score (median^a^)	−2.77 (−3.01 to −2.48)	−2.35 (−2.48 to −1.98)	−1.94 (−2.25 to −1.66)	−1.28 (−1.51 to −1.00)	<0.001
Milan criteria, beyond (%)	89 (31.4%)	26 (49.1%)	48 (63.2%)	18 (60.0%)	<0.001
Positive for MVI (%)	16 (5.7%)	3 (5.7%)	13 (17.1%)	9 (30.0%)	0.017
Positive for EHM (%)	13 (4.6%)	2 (3.8%)	11 (14.5%)	4 (13.3%)	0.007
MVL (%)	28 (9.9%)	20 (37.7%)	36 (47.4%)	21 (70.0%)	<0.001
GNRI score (median^a^)	106.8 (102.7–112.1)	95.1 (94.0–96.4)	88.2 (86.1–90.1)	77.1 (72.8–79.8)	<0.001

Abbreviations: ECOG PS, Eastern Cooperative Oncology Group Performance Status; EHM, extra‐hepatic metastasis; GNRI, geriatric nutritional risk index; HBV, hepatitis B virus; HCV, hepatitis C virus; mALBI, modified albumin–bilirubin; MVI, major vessel invasion; MVL, muscle volume loss.

^a^ Median values in parentheses show interquartile range.

**Figure 1:**
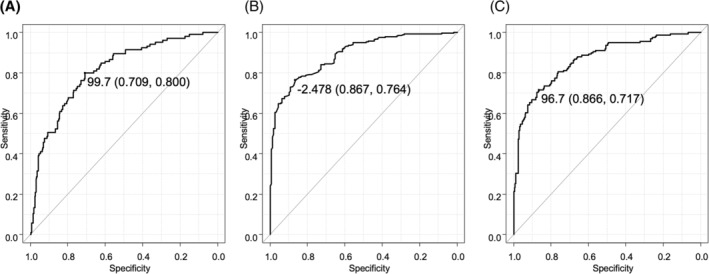
Cut‐off values of geriatric nutritional risk index (GNRI) score and albumin–bilirubin (ALBI) score for muscle volume loss, and that of GNRI for modified ALBI grade 2b. The cut‐off vale for GNRI score was 99.7 (specificity/sensitivity = 0.709/0.800) (area under the curve [AUC] 0.813, 95% confidence interval [CI]: 0.766 to 0.859) (A). The cut‐off ALBI score for GNRI mild decline status (score < 98) was −2.478 (specificity/sensitivity = 0.867/0.764) (AUC 0.892, 95% CI: 0.863 to 0.921) (B), whereas that of GNRI score for modified ALBI grade 2b (score > −2.27) was 96.7 (specificity/sensitivity = 0.866/0.717) (AUC 0.867, 95% CI: 0.831 to 0.903) (C).

The cut‐off ALBI score for MVL was −2.093 (specificity/sensitivity = 0.783/0.448) (AUC 0.636, 95% confidence interval [CI]: 0.574 to 0.698) (*Figure* *S1*), whereas the cut‐off GNRI score for MVL was 99.7 (specificity/sensitivity = 0.709/0.800) (AUC 0.813, 95% CI: 0.766 to 0.859) (*Figure* *1* *A*). The cut‐off GNRI score for predicting MVL was 99.7 (specificity/sensitivity = 0.730/0.795) (AUC 0.824, 95% CI: 0.771 to 0.878) in males and 99.4 (specificity/sensitivity = 0.685/0.781) (AUC 0.782, 95% CI: 0.689 to 0.876) in females.

Sub‐analysis results showed a significant relationship between GNRI and ALBI scores (*r* = −0.738, 95% CI: −0.778 to −0.692, *P* < 0.001) (*Figure* *S2*). The cut‐off ALBI score for GNRI mild decline status (score < 98) was −2.478 (specificity/sensitivity = 0.867/0.764) (AUC 0.892, 95% CI: 0.863 to 0.921) (*Figure* *1* *B*) and that for mALBI grade 2b (score > −2.27) was 96.7 (specificity/sensitivity = 0.866/0.717) (AUC 0.867, 95% CI: 0.831 to 0.903) (*Figure* *1* *C*). For patients without ascites, the cut‐off ALBI score for MVL was −2.650 (specificity/sensitivity = 0.507/0.692) (AUC 0.604, 95% CI: 0.533 to 0.676) (*Figure* *S3* *a*), whereas the cut‐off GNRI score for MVL was 99.7 (specificity/sensitivity = 0.760/0.744) (AUC 0.803, 95% CI: 0.747 to 0.858) (*Figure* *S3* *b*). After exclusion of patients beyond the Milan criteria, the cut‐off ALBI score for MVL was −2.62 (specificity/sensitivity = 0.577/0.608) (AUC 0.598, 95% CI: 0.510 to 0.686), whereas the cut‐off GNRI score for MVL was 103.5 (specificity/sensitivity = 0.639/0.863) (AUC 0.810, 95% CI: 0.745 to 0.875). Additionally, the cut‐off GNRI score for MVL in HCC patients beyond the Milan criteria was 94.5 (AUC 0.808, 95% CI: 0.737 to 0.878).

## Discussion

The present results showed that the frequency of MVL, which has been defined as pre‐sarcopenia,^16^ increased as nutritional status (GNRI) worsened (*P* < 0.001). Although the GNRI was originally created for assessing geriatric nutritional status, the present study was conducted under the consideration that it also reflects the effects of muscle loss. When the cut‐off GNRI score for predicting MVL was analysed according to gender, those values were approximated (males 99.7, females 99.4). The GNRI uses different formulas for calculating standard weight for males and females, which may have contributed to those results. Thus, the cut‐off GNRI score for MVL was 99.7 (approximately equal to the cut‐off value for GNRI mild decline) in all patients, with the same score found in patients without ascites. For the GNRI normal status patients with MVL (28/283: 9.9%), that was thought to be mainly due to aging, because those with MVL were older (77 vs. 72 years, *P* = 0.006).

Recently, decreased muscle has been commonly reported as a complication in CLD patients.^17^ Hanai et al. noted a hazard ratio (HR) of mortality from sarcopenia of 3.03 (95% CI: 1.42 to 6.94)^18^ and, in another study, found that LC patients showed a muscle volume decline of −2.2%/year.^19^ It is important to assess sarcopenia, especially in cases of LC, because the HR for mortality of LC patients in accordance with muscle mass was found to be 0.78 (95% CI: 0.68 to 0.89, *P* < 0.001), implying that mortality decreases at a rate of 22% in cases with higher muscle mass.^20^ Moreover, MVL has also been described as a prognostic factor for recurrence after curative treatments (HR 1.77, *P* < 0.001), as well as overall survival (OS) in HCC patients treated with either curative (HR 2.152, *P* < 0.001) or palliative (HR 2.358, *P* < 0.001) procedures.^5^

As noted above, an evaluation of MVL has clinical importance, though an important issue is that the assessment requires special expensive equipment, such as BIA or CT, and/or subjecting the patient to X‐ray exposure. Previously, a finger‐circle (*yubi‐wakka*) test using the patient's own fingers was reported as an easy to perform tool for assessment of the early stage of MVL in CLD patients,^21^ though it is thought to be difficult for evaluation of relative changes in nutritional status. Therefore, the results presented here indicate that GNRI might be a predictive tool for MVL in CLD patients that is easy to use in clinical situations. When GNRI assessment of a CLD patient shows a decline that is mild or greater, the clinician should keep in mind the assessment of muscle volume along with routine nutritional intervention^22^ with a goal to maintain daily activities of the patient^23^ to prevent progression of sarcopenia.

Immune checkpoint inhibitors (ICIs) have recently been developed and shown to have a great role in cancer treatment. Meta‐analysis findings of patients treated with an ICI showed that MVL was related with poor objective response rate (ORR) (OR 0.46, 95% CI: 0.28 to 0.74, *P* = 0.001), disease control rate (DCR) (OR 0.44, 95% CI: 0.31 to 0.64, *P* < 0.0001), progression‐free survival (PFS) (HR 1.46, 95% CI: 1.20 to 1.78, *P* = 0.0001) and OS (HR 1.73, 95% CI: 1.36 to 2.19, *P* < 0.0001).^24^ Furthermore, also in patients who received atezolizumab plus bevacizumab treatment for unresectable HCC, MVL was found to be a prognostic factor related to PFS (HR 1.479, 95% CI: 1.020 to 2.144, *P* = 0.039) and OS (HR 2.119, 95% CI: 1.150 to 3.904, *P* = 0.016).^25^ These results indicate that MVL is also an important prognostic factor in the current treatment of HCC. Therefore, it is important to evaluate MVL in HCC patients. In our results, the cut‐off value of GNRI for MVL was 99.7, which was approximate for the cut‐off for GNRI mild decline, and a cut‐off ALBI score for predicting GNRI mild decline was −2.478 (AUC 0.892, 95% CI: 0.863 to 0.921). That cut‐off value for GNRI mild decline status is near the middle of mALBI grade 2a, whereas the cut‐off value for GNRI for predicting mALBI grade 2b was 96.7 (AUC 0.867, 95% CI: 0.831 to 0.903), approximating that for the upper range of mild decline status (GNRI 98). Together, these results suggest that hepatic reserve function and nutritional status are closely related in CLD patients with HCC. Thus, it is suggested that nutritional status begins to deteriorate when ALBI grade 1 changes to 2a, whereas GNRI mild decline status likely has become established by the time the patient reaches mALBI grade 2b.

Based on our results, GNRI is considered to be a useful predictor for MVL in CLD patients. However, this study has some limitations. First, this was a single‐centre study conducted in a retrospective manner. Second, all the subjects were HCC patients. Third, there were no data related to muscle strength such as handgrip strength available for the present cohort. Finally, the relationship between relative changes in GNRI score and muscle volume in each patient was not assessed. To obtain concrete conclusions, a multicentre study is needed with a larger number of CLD patients without HCC.

In conclusion, the present findings show GNRI to be an easy and possibly effective prediction tool for MVL in CLD patients. To maintain a normal GNRI, nutritional intervention is thought to be important and muscle volume should be assessed when an abnormal GNRI value is demonstrated.

## Supporting information


**Table S1.** Clinical characteristics of GNRI normal status patients with and without MVL (*n* = 283).

**Figure S1.** Cut‐off values for ALBI for muscle volume loss in all 442 patients. The cut‐off albumin‐bilirubin (ALBI) score for muscle volume loss (MVL) was −2.093 (specificity/sensitivity = 0.783/0.448) (AUC 0.636, 95% CI: 0.574–0.698).

**Figure S2.** Relationship of ALBI with GNRI in all 442 patients. There was a significant relationship between albumin‐bilirubin (ALBI) and geriatric nutritional risk index (GNRI) score (*r* = −0.738, 95% CI: −0.778 to −0.692, *P* < 0.001)

**Figure S3.** Cut‐off values for ALBI and GNRI scores for muscle volume loss in patients without ascites (*n* = 370). After excluding patients with ascites, the cut‐off albumin‐bilirubin (ALBI) score for muscle volume loss (MVL) was −2.650 (specificity/sensitivity = 0.507/0.692) (AUC 0.604, 95% CI: 0.533–0.676) (a), while the cut‐off geriatric nutritional risk index (GNRI) score for MVL was 99.7 (specificity/sensitivity = 0.760/0.744) (AUC 0.803, 95% CI: 0.747–0.858) (b).
