# Supplementary material for: Simple method for predicting muscle volume loss using geriatric nutritional risk index in hepatocellular carcinoma patients
Source: J Cachexia Sarcopenia Muscle. 2023 May 19;14(4):1906–11. doi: 10.1002/jcsm.13268 (PMC10401522; doi:10.1002/jcsm.13268)
Supplement: Supplementary file 1 — Table S1. Clinical characteristics of GNRI normal status patients with and without MVL (n = 283). [file JCSM-14-1906-s002.docx]

Supplemental Table 1. Clinical characteristics of GNRI normal status patients with and without MVL (n=283)

|  | Negative for MVL (n=255) | Positive for MVL (n=28) | P value |
| --- | --- | --- | --- |
| Age, years (median*) | 72 (67-79) | 77 (71-83) | 0.006 |
| Gender, male (%) | 65 (25.5%) | 10 (35.7%) | 0.262 |
| Body mass index, kg/m2 (median*) | 25.1 (23.3-27.4) | 21.9 (20.0-23.3) | <0.001 |
| ECOG PS, 0:1:2:3:4 | 224:17:9:3:2 | 20:7:1:0:0 | 0.044 |
| Etiology (HCV:HBV:HCV&HBV:alcohol:others) | 105:22:1:49:78 | 8:2:0:6:12 | 0.536 |
| Child-Pugh class, A:B:C | 243:10:2 | 28:0:0 | 0.680 |
| Ascites, none:controllable:massive | 236:14:5 | 27:1:0 | 1.000 |
| mALBI grade, 1:2a:2b:3 | 166:45:42:2 | 23:4:1:0 | 0.235 |
| ALBI score (median*) | -2.77 (-2.99 to -2.45) | -2.89 (-3.10 to -2.73) | 0.023 |
| Milan criteria, beyond (%) | 80 (31.4%) | 9 (32.1%) | 1.000 |
| Positive for MVI (%) | 16 (6.3%) | 0 (0%) | 0.382 |
| Positive for EHM (%) | 11 (4.3%) | 2 (7.1%) | 0.375 |

*Median values in parentheses show interquartile range.
