# Supplementary figures and images for: Simple method for predicting muscle volume loss using geriatric nutritional risk index in hepatocellular carcinoma patients
Source: J Cachexia Sarcopenia Muscle. 2023 May 19;14(4):1906–11. doi: 10.1002/jcsm.13268 (PMC10401522; doi:10.1002/jcsm.13268)

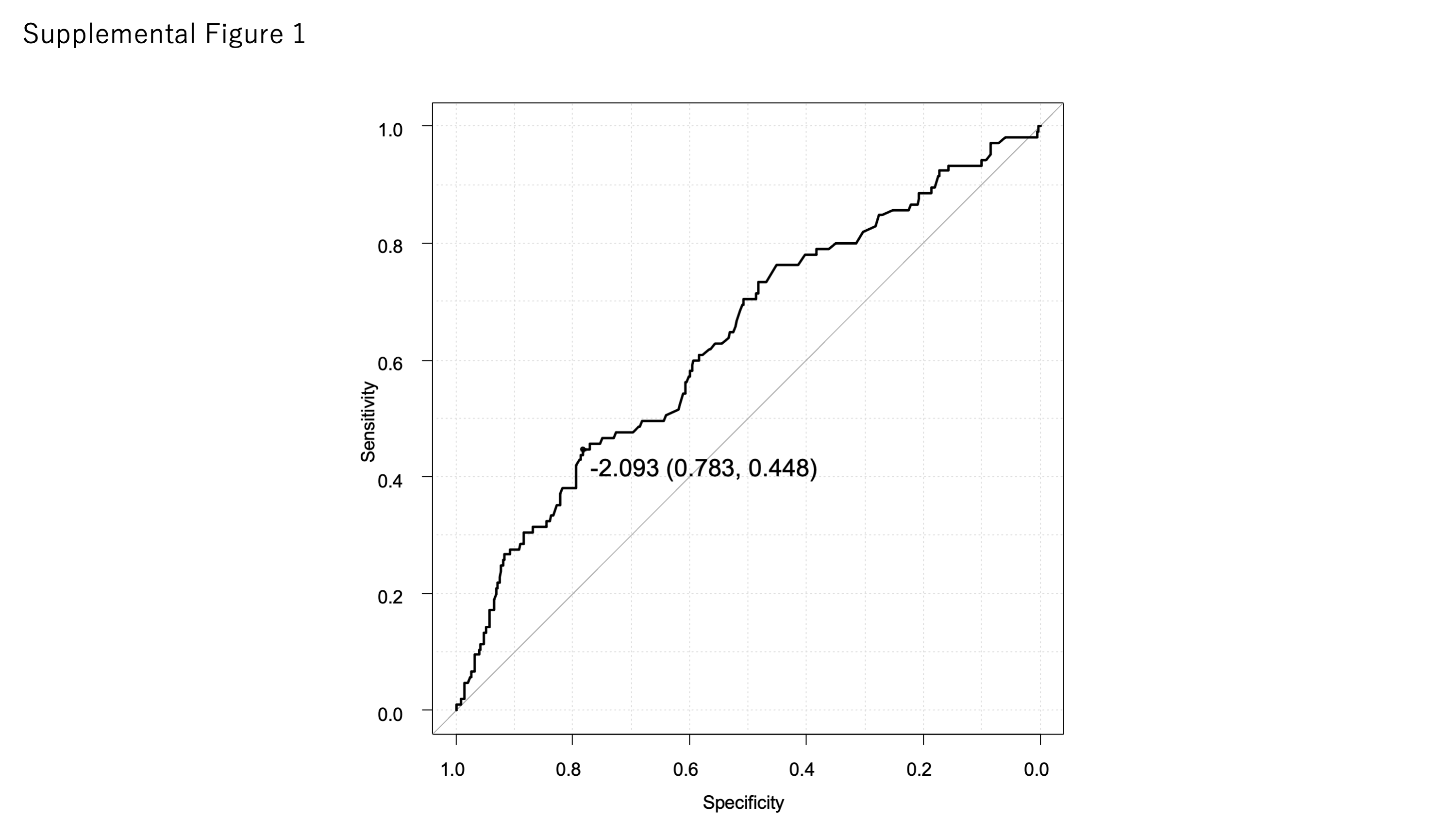

Supplement: Supplementary file 2 — Figure S1. Cut‐off values for ALBI for muscle volume loss in all 442 patients. The cut‐off albumin‐bilirubin (ALBI) score for muscle volume loss (MVL) was −2.093 (specificity/sensitivity = 0.783/0.448) (AUC 0.636, 95% CI: 0.574–0.698). [file JCSM-14-1906-s004.tiff]

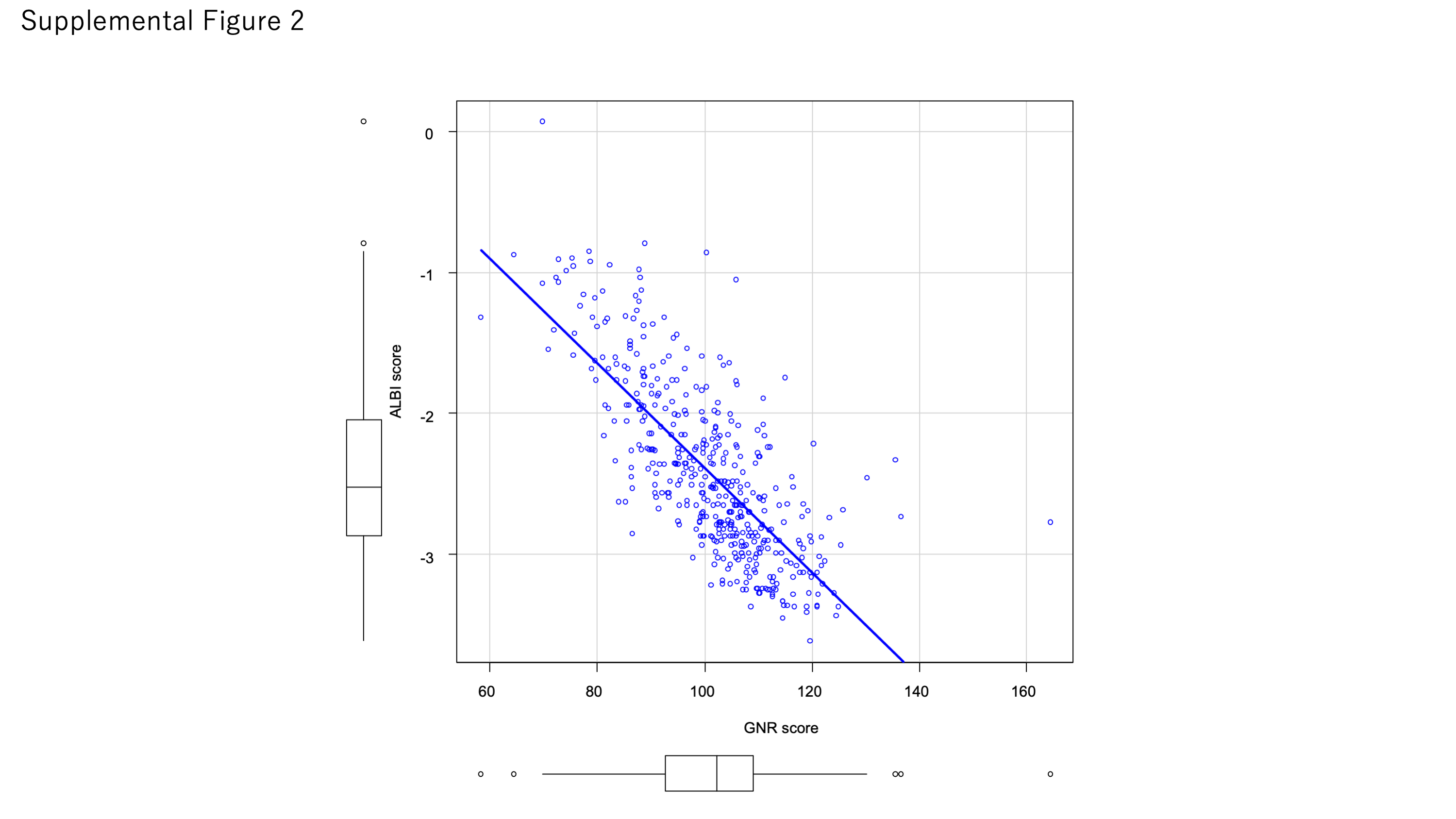

Supplement: Supplementary file 3 — Figure S2. Relationship of ALBI with GNRI in all 442 patients. There was a significant relationship between albumin‐bilirubin (ALBI) and geriatric nutritional risk index (GNRI) score (r = −0.738, 95% CI: −0.778 to −0.692, P < 0.001) [file JCSM-14-1906-s003.tiff]

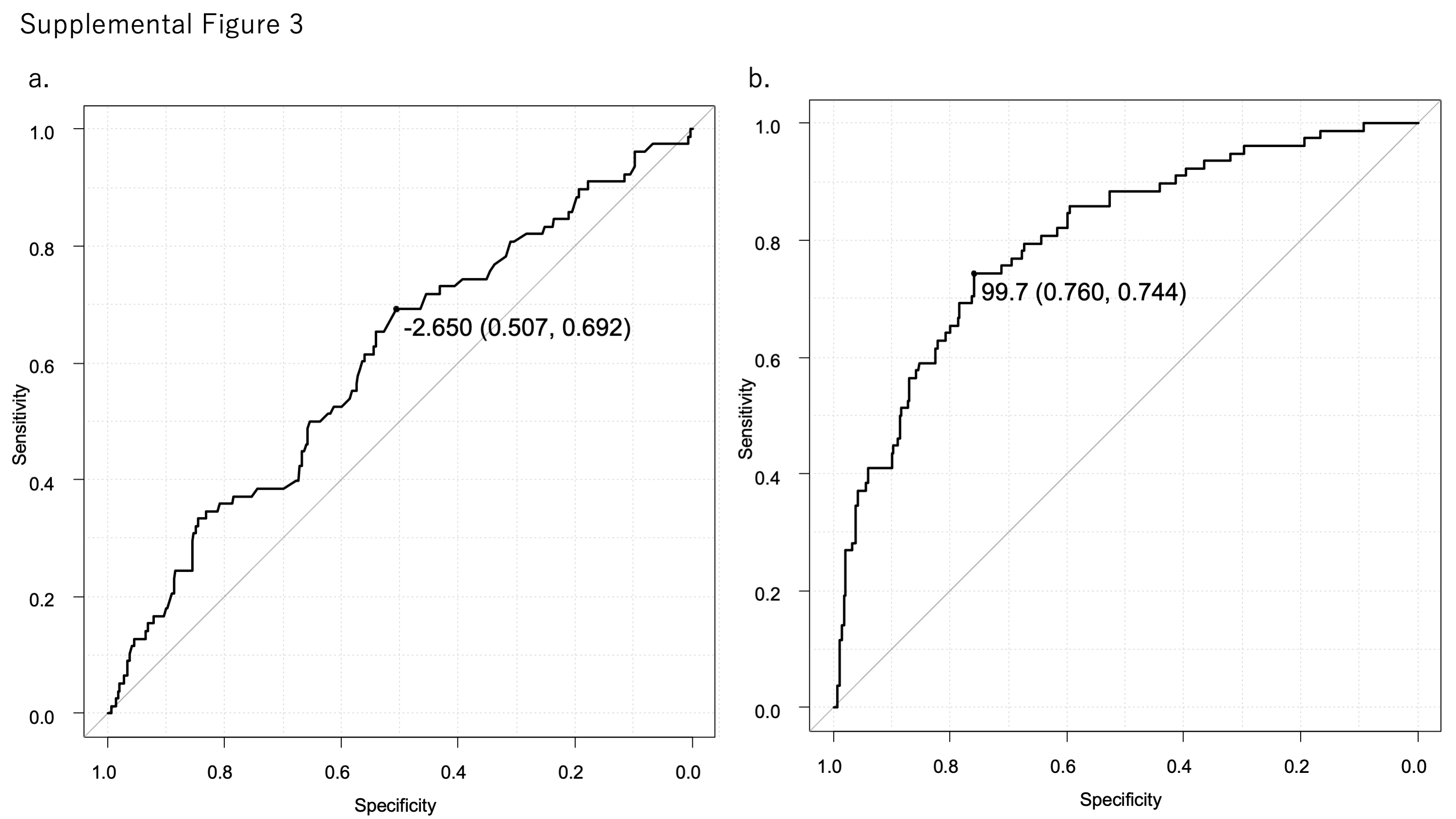

Supplement: Supplementary file 4 — Figure S3. Cut‐off values for ALBI and GNRI scores for muscle volume loss in patients without ascites (n = 370). After excluding patients with ascites, the cut‐off albumin‐bilirubin (ALBI) score for muscle volume loss (MVL) was −2.650 (specificity/sensitivity = 0.507/0.692) (AUC 0.604, 95% CI: 0.533–0.676) (a), while the cut‐off geriatric nutritional risk index (GNRI) score for MVL was 99.7 (specificity/sensitivity = 0.760/0.744) (AUC 0.803, 95% CI: 0.747–0.858) (b). [file JCSM-14-1906-s001.tiff]
